# Bone Bruise Patterns in Ligamentous Injuries of the Knee With Focus on Anterior Cruciate Ligament

**DOI:** 10.7759/cureus.32113

**Published:** 2022-12-01

**Authors:** Sanjay Yadav, Raghul Dhakshanamoorthy, Ishan Kumar, Adity Prakash, Ramya Nagarajan

**Affiliations:** 1 Orthopedics, Banaras Hindu University, Varanasi, IND; 2 Radiology, Banaras Hindu University, Varanasi, IND; 3 Epidemiology and Public Health, National Institute of Epidemiology, Chennai, IND

**Keywords:** cruciate, ligament, bruise, tibial condyle, femoral condyle, mri, bone

## Abstract

Introduction

After sustaining an anterior cruciate ligament (ACL) injury, the bone bruises seen on magnetic resonance imaging (MRI) could reveal plenty of information regarding the loading mechanisms causing injury to the ACL. The current study was conducted to evaluate the common distribution patterns of bone bruises following an ACL injury and understand the loading mechanisms.

Methods

The knee MRI sequences of the patients operated arthroscopically for an injured ACL between August 2016 to August 2018 were selected for the study. The distribution pattern of the bone bruises was determined using the sagittal and coronal sections of MRI. The pattern of distribution of the bone bruises was categorized and analyzed by two independent observers.

Results

Twenty-two patients were found to have bone bruises diagnosed in the MRI scans. The mean age of the patients was 27.8 ± 8.7 years. The pattern of a bone bruises in only the lateral femoral and tibial compartments was the most typical pattern observed in this study. The study pattern has a significant anterior distribution of bone bruises on the outer (lateral) compartment of both the femur and tibia as compared to the inner (medial) compartment (p< .05 and p > .05, respectively). The inter-rater reliability between the two observers by Cronbach's Alpha was 93.2%.

Conclusion

Having the appropriate information regarding the pattern distribution of bone bruises and the concomitant injuries associated with it furthers our knowledge and helps us understand the loading mechanisms of ACL tears. A combination of coup forces acting on the lateral compartment and the contrecoup varus force on the medial compartment of the knee during the primary pivot-shift injury suggests an an involvement of multiplanar loading patterns at the point of sustaining ACL tear.

## Introduction

An anterior cruciate ligament (ACL) tear is one of the knee's most frequent and catastrophic sports injuries. Obtaining an understanding and awareness regarding the mechanisms of ACL loading and identifying the factors that pose a risk of injury is of the utmost importance for implementing and enhancing preventive measures [1]. Following an ACL injury, the femoral and tibial bone bruises visualized on an MRI have the potential to reveal important information regarding the mechanisms of an ACL tear. A bone bruise is a traumatically sustained bone contusion showing low and high signal intensities on T1- and T2-weighted scans, respectively [2].

The current study evaluated the common distribution patterns of bone bruising following ACL injury. The hypothesis was that bruising on only the lateral compartments of the femur and tibia would be the most frequent bruise pattern. Bone bruises are currently believed to provide insight into the mechanisms of ACL injury by leaving a footprint of the impact when the injury occurs [3]. In a few studies, concerns have been raised about more severe damage to the knee joint affecting the degenerative process, which could affect the long-term clinical outcomes in such cases. Bone bruises are also reported to be associated with increased pain in such cases. Bone bruise severity may also signify associated inflammation that needs to settle before any surgical intervention.

Using preoperative MRI scans, Costa-Paz et al. derived a three-level grading system depending on the bone bruise location and appearance. In Type I bruising, the signal is diffuse with medullary changes, while in Type II bruising, the signal is localized and closer to the adjacent articular surface. Finally, Type III involves a disrupted typical contour of the cortical surface [4]. Restoring knee stability and function is crucial to the success of ACL reconstruction, although the long-term success of ACL reconstruction also depends upon preventing degenerative changes. Song et al. studied bruising bone following non-contact-induced acute ACL injury. In this case, the bruising configuration most often involved the outer tibial compartment (73.1%), followed by bruising to the outer femoral compartment (60.6%) [5]. 

## Materials and methods

Patients who visited our institution between August 2016, and August 2018, after sustaining ACL injury managed by ACL reconstruction, were identified, and their knees (on the MRI side) were used in this study. The institutional ethics clearance was obtained (IEC/IMS/2100). The demographic characteristics and data concerning the bone bruise configurations and the concomitant associated injuries on the MRI were documented. Scans were carried out using a 1.5-T scanner with a 5-mm thickness and 256 × 256 matrices in a three-dimensional plane. The bone bruise configuration in the anterior-posterior direction was determined by reviewing the MRI sequences in the sagittal plane. In contrast, the lateral-medial direction was assessed based on the sequences in the coronal plane. The presence or absence and intensity of bone bruises were recorded, and the intra-compartmental distribution of the bruises was documented. The bone bruise distribution pattern was categorized and analyzed by two independent observers.

Detailed information about the injuries was obtained by reviewing the clinical case notes, operative notes, and imaging sequences. The injury mechanism- direct or indirect; and any associated concomitant articular or non-articular injuries were confirmed by an experienced radiologist and orthopedic surgeon through MRI and arthroscopic surgery, respectively. The radiology was analyzed independently by one observer of radiology and one observer of orthopedics to establish inter-observer reliability. The inclusion criteria included: Having an arthroscopically confirmed ACL tear; Having an MRI not more than 6 to 8 weeks old from the day of trauma; Being within the age range of 18 to 40 years.

The exclusion criteria included: Having a previous injury to the ipsilateral knee; having an injury to the posterior cruciate ligament; having an injury to the collateral ligaments (medial and lateral), and having a poor-quality MRI scan.

The MRI knee scans were first categorized according to whether the bone bruises were present. The compartmental distribution of bone bruises was independently noted in the lateral-medial direction, followed by the anterior-posterior direction. The patients' demographic parameters were described using counts and percentages. The continuous variables' means and standard deviations (SDs) are presented. The placement of the lateral and medial compartments in the anterior-posterior direction was compared using a paired t-test. A paired t-test was also used to compare the bone bruise configuration in the anterior-posterior plane between the outer (lateral) and inner (medial) compartments.

The severity of the bone bruises was compared between the lateral and medial compartments based on signal strength for each participant's imaging sequence. In terms of notation, ML indicates the bone bruise configuration in the lateral-medial plane, ranging from 0% to 100% (0 and 100 indicating the closest proximity to the lateral and medial sections, respectively). Similarly, AP indicates the location of the bone bruise configuration anteroposteriorly, ranging from 0% to 100% (0 and 100 indicating the closest proximity to the anterior and posterior compartments of the bone, respectively). Cronbach's alpha calculated the inter-rater reliability between the two observers. A cut-off rate of 0.05 was used to indicate statistical significance (Type I error). All the statistical analyses were carried out using SPSS Version 16 (IBM).

## Results

Among the 33 patients included in the study, only 22 were found to have bone bruises on their MRI scans. The mean ± SD ages of the patients with and without bone bruises were 27.8 ± 8.7 and 28.6 ± 8.0, respectively (Table 1).

**Table 1:** Frequency distribution (%) of males and females in cases with a bruise and without a bruise.

Group	N	Age (in years)	Gender- n (%)	
		Mean ± sd	Male	Female
Bruise	22	27.8 ± 8.7	21 (95.5%)	1 (4.5%)
No bruise	11	28.6 ± 8.1	11 (100%)	0 (nil)

Among the 22 patients with bone bruising, 95.5% (21) were male, and 4.5% (1) were female, whereas 100% of the patients without bone bruises were male.

The distribution patterns of the bone bruises in the current study per the orthopedic observer include bruises detected on: The outer compartment of both the femur and the tibia (LF & LT); The outer compartment of the femur and the inner compartment of the tibia (LF & MT); The inner compartment of the femur and either the outer or inner compartment of the tibia (MF &BT); The inner compartment of the femur and the outer compartment of the tibia (MF & LT). Numerous configurations, such as bruising on the inner compartments of either of the femur and tibia (MF & MT), on the inner and outer compartments of the tibia (MT & LT), on the medial tibia (MT), on the lateral tibia (LT), and the lateral femur (LF), were also observed. The LF &LT pattern was the most common configuration, followed by LF & LT and LF (Table 2).

**Table 2:** Frequency n (%) distribution of bone bruise location in evaluation by radiologist and orthopaedic specialist. M- medial; L- lateral; F- femur; T- tibia

Location	Radiologist n (%)	Orthopedics n (%)
BF & BT	1 (3%)	
BF & LT	-	-
LF	1 (3%)	3 (9.1%)
LF & LT	6 (18.2%)	7 (21.2%)
LF & MT	1 (3%)	3 (9.1%)
LT	4 (12.1%)	1 (3%)
MF &LT	1 (3%)	2 (6.1%)
MF & MT	3 (9.1%)	2 (6.1%)
MF & LT	1 (3%)	-
MT	1 (3%)	1 (3%)
MT & LT	-	2 (6.1%)
MF & MT & LT		1 (3%)
MF	2 (6.1%)	
MF & BT	2 (6.1%)	
No bruise	10 (30.3%)	11 (33.3%)
Total	33 (100.0%)	33 (100.0%)

**Figure 1:**
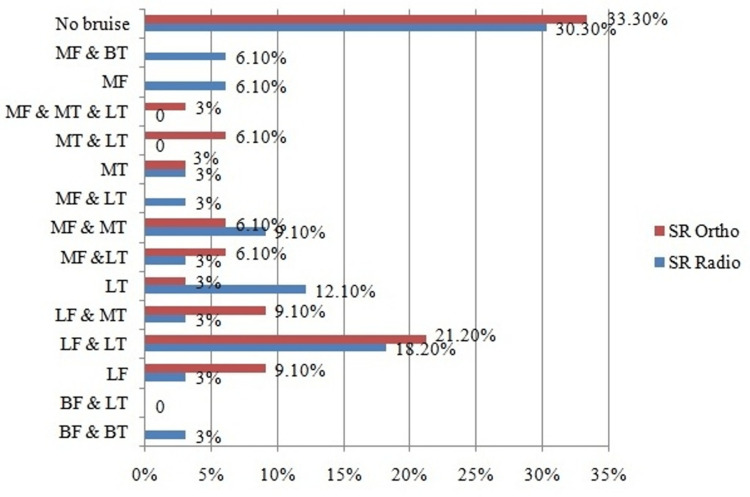
Frequency (%) distribution of location of bone bruise by orthopedics and radiology specialists M- medial; L- lateral; F- femur; T- tibia

The precise tibial and femoral bone bruise configurations are depicted in both dimensional planes, i.e., lateral-medial and anterior-posterior (Figure 1).

One radiologist and one orthopedic specialist analyzed the MRI scans independently to visualize the bone bruises and their placement in the lateral-medial and anterior-posterior planes to establish inter-observer reliability (using Cronbach's alpha).

The gray values on the outer compartment of the tibia and femur were significantly higher than those on the medial compartment of the tibia and femur. The gray values indicate the brightness of a pixel on MRI imaging, which appears to be a valid method of differentiating the signal intensities. The paired t-test revealed that the bone bruises on the outer femoral and tibial compartment (femur: 47.5% ± 20.4%; tibia: 65.1% ±18.0%) were concentrated further anterior than those on the medial compartment (femur: 49.0%±24.1%; tibia: %: 52.4 ± 25.6; p<0.05 and p >0.05, respectively) (Table 3).

**Table 3:** Comparison of mean % of bone bruise location by paired t-test. M- medial condyle; L- lateral condyle

	N	Mean % ± sd (%)	Diff. Mean ± SEM (%)	p-value
Tibia M	14	52.4 ± 25.6	3.4 ± 29.7	0.6
Femur M	14	49.0 ± 24.1		
Tibia L	13	65.1 ± 18.0	17.6 ± 22.9	0.01*
Femur L	13	47.5 ± 20.4		
Femur M	16	50.1 ± 23.2	1.3 ± 29.1	0.8
Femur L	16	48.7 ± 19.1		
Tibia L	18	64.6 ± 17.7	16.5 ± 31.0	0.03*

**Figure 2:**
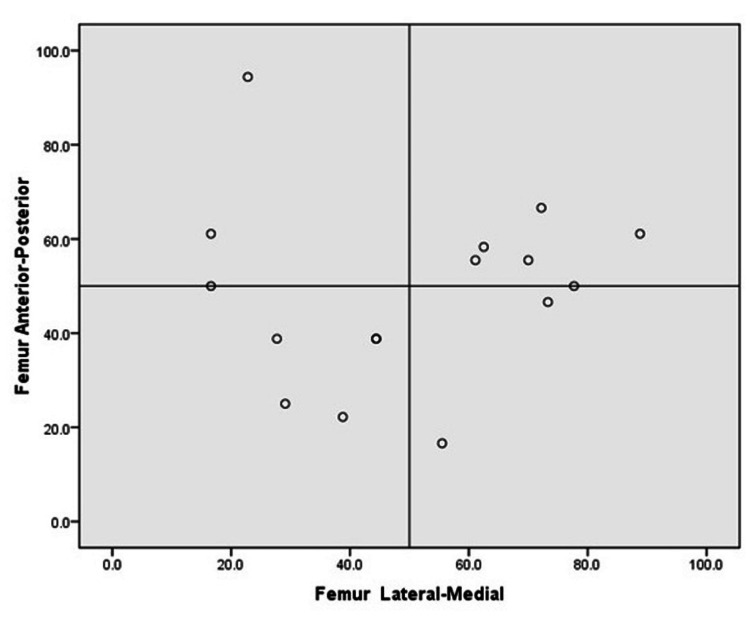
Distribution of bone bruise in femur: lateral-medial and anterior-posterior directions

**Figure 3:**
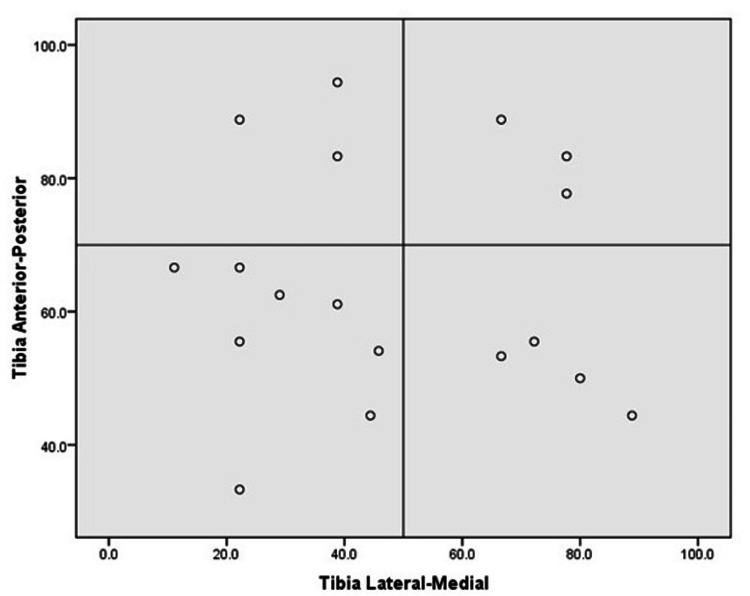
Distribution of bone bruise in tibia lateral-medial and anterior-posterior directions

A significant difference was observed between the distribution pattern of the bone bruises on the lateral (64.6% ±17.7%) and medial compartments (48.1%±24.7%) of the tibia (p<0.05). No significant difference was observed in the bone bruise pattern of the lateral (47.5% ±20.4%) and medial compartments (49.0% ± 24.1%) of the femur (p >0.05). The inter-rater reliability between the orthopedic specialist and radiologist based on Cronbach's alpha was 93.2%, indicating excellent agreement between the two observers. Figure 2 shows the distribution of bone bruise locations on the femur in the lateral-medial and anterior-posterior directions. Figure 3 shows the distribution of bone bruises on the tibia in the lateral-medial and anterior-posterior directions.

## Discussion

Our results point towards bruising on only the outer femoral and tibial compartments (21%, N=33) being the most common pattern observed, which has the potential to help us better understand ACL injury and its loading patterns. In particular, this result can be explained by a knee pivot-shift valgus collapse injury [6-9]. Previous investigators have shown that bone bruises on the lateral compartment have a larger volume than medial-compartment bruises [10]. The results of the current study also revealed other common patterns, such as the LF & MT and LF configurations.

Huijuan et al. studied 207 patients and found the most common configurations to be bruises on the lateral femoral and lateral tibial compartments (44.4%), followed by bruising on the outer and inner compartments of both the femur and tibia (29.0%) [10]. In a study of 168 patients by Palanisamy et al., the vast majority (92.3%) of patients exhibited bone contusions on their MRI. Among these, the bone contusions on the outer tibial compartment (83.9%) were the most frequent, followed by those on the outer femoral compartment (78.6%). Although less frequent in the inner tibial compartment (56.5%), the lowest number was in the inner femoral compartment (29.8%) [11].

The results of the current study indicate that the LF & LT pattern presented by the patients with ACL injury was caused by the brunt forces acting on the outer femoral and tibial compartments. Wittstein et al. reported outer-compartmental bone bruises of the tibia to be more severe than inner-compartmental ones in most patients, indicating severe brunt forces acting on the lateral side [12]. This pattern could be the result of the coup forces acting on the lateral compartment resulting from anterior translation of the tibia during the primary pivot-shift self-reduction along with the contrecoup varus malalignment force acting on the medial side of the knee (because the primary pivot-shift hit causes self-reduction of the knee)[13]. Overall, these results indicate the involvement of multiplanar loading patterns during an ACL injury.

As per the results of our study, tibial bruise configurations were found to be more posterior, while femoral configurations were found to occupy the anterior or middle (central) compartment. This suggests that the anterior femoral compartment impacts the posterior tibial compartment, further indicating the possibility of anterior translation of the tibia with respect to the femur [14,15]. Further data obtained in this study demonstrated a significant anterior distribution of bone bruises on the outer femoral and tibial compartments compared with that on the inner compartment. If the knee was in minimal flexion when the injury was sustained, the bone bruise was found more towards the anterior compartment of the lateral femoral condyle. When the knee was in moderate flexion at an injury, a posteriorly located contusion of the femoral condyle was observed. In both scenarios, the bone bruise was located on the posterior section of the tibial plateau.

Since the ACL provides central restraint to the shear forces acting anteriorly, a huge amount of shear force can be produced on the tibia anteriorly by a similar degree of anterior tibial translation. Large anterior translation of the tibia could produce large anterior tibial shear forces, representing a key risk factor for ACL injury [16]. In a report by McCauley et al., two independent radiologists examined MR sequences of 39 people with ACL tears operated on arthroscopically. For the two reviewers, the presence of bone bruises in the posterolateral tibial plateau had a sensitivity of 50% and 46%, respectively, with corresponding specificities of 97%. They concluded that the visualization of bone bruises on the posterolateral tibial plateau predicted ACL tear with high specificity and that its mere presence should be regarded as solid evidence of ACL tear [17]. In our study, the inter-rater reliability between the two independent observers based on Cronbach's alpha was 93.2%, representing the excellent agreement between the radiologist and orthopedic specialist.

Avoiding extreme valgus loading while engaging in sports activities must be emphasized in programs that prevent ACL injuries. Assuming that medial compartmental bone bruises occur due to contrecoup injuries, the LF & LT pattern could be considered a direct and precise footprint occurring after the injury and thus could provide many valuable clues regarding the loading mechanisms of ACL injury. Future studies on ACL injury mechanisms investigating the distribution pattern of bone bruises should emphasize MRIs with a single bone bruise on the tibia and femur.

This study had several limitations. First, the study population was less ideal, especially the proportion of women. Second, since the non-contact-injury patients had severe bone bruises compared to the contact group patients, more studies are required to compare the configuration patterns of bone bruises between these groups.

## Conclusions

Bruising on the outer femoral and tibial compartments was the most common pattern observed in this study. Outer-compartmental bone bruises were also found to be more severe than inner-compartmental ones, indicating anterior tibial translation with respect to the femur following a torn ACL. Long-term prospective studies are required to understand the natural history of bone bruises better and suggest specific target treatments to cover the findings on deranged joints associated with ACL lesions.
